# Trends in mouth cancer incidence in Mumbai, India (1995–2009): An age-period-cohort analysis

**DOI:** 10.1016/j.canep.2016.03.007

**Published:** 2016-06

**Authors:** Krithiga Shridhar, Preetha Rajaraman, Shravani Koyande, Purvish M. Parikh, Pankaj Chaturvedi, Preet K. Dhillon, Rajesh P. Dikshit

**Affiliations:** a Centre for Chronic Conditions and Injuries, Public Health Foundation of India, 4th Floor, Plot No. 47, Sector 44, Gurgaon 122002, Haryana, India; b Centre for Global Health, National Cancer Institute, Bethesda, USA; c Mumbai Cancer Registry, 74, Jerbai Wadia Road, Bhoiwada, Parel, Mumbai 400 012, India; d Head and Neck Surgery, Tata Memorial Hospital, Dr. E Borges Road, Parel, Mumbai 400012, India; e Centre for Cancer Epidemiology, Tata Memorial Hospital, Dr. E Borges Road, Parel, Mumbai 400 012, India

**Keywords:** Mouth cancer, Cancer trend, India, Age-period-cohort analysis, Cancer registry, Age-specific rate, Age standardized rate, Risk factors, Mumbai, Net drift

## Abstract

**Introduction:**

Despite tobacco control and health promotion efforts, the incidence rates of mouth cancer are increasing across most regions in India. Analysing the influence of age, time period and birth cohort on these secular trends can point towards underlying factors and help identify high-risk populations for improved cancer control programmes.

**Methods:**

We evaluated secular changes in mouth cancer incidence among men and women aged 25–74 years in Mumbai between 1995 and 2009 by calculating age-specific and age-standardized incidence rates (ASR). We estimated the age-adjusted linear trend for annual percent change (EAPC) using the drift parameter, and conducted an age–period–cohort (APC) analysis to quantify recent time trends and to evaluate the significance of birth cohort and calendar period effects.

**Results:**

Over the 15-year period, age-standardized incidence rates of mouth cancer in men in Mumbai increased by 2.7% annually (95% CI:1.9 to 3.4), p < 0.0001) while rates among women decreased (EAPC = −0.01% (95% CI:−0.02 to −0.002), p = 0.03). APC analysis revealed significant non-linear positive period and cohort effects in men, with higher effects among younger men (25–49 years). Non-significant increasing trends were observed in younger women (25–49 years).

**Conclusions:**

APC analyses from the Mumbai cancer registry indicate a significant linear increase of mouth cancer incidence from 1995 to 2009 in men, which was driven by younger men aged 25–49 years, and a non-significant upward trend in similarly aged younger women. Health promotion efforts should more effectively target younger cohorts.

## Highlights

• We conducted an age-period-cohort (APC) analysis of mouth cancer in Mumbai, India.
• An overall upward trend was observed in 1995–2009 for men but not women.
• The increase was mainly in younger men (aged 25–49 years).
• A suggestive increase was also seen in younger women (aged 25–49 years).
• Our data suggest a need for focused tobacco control efforts in younger cohorts.

## 1 Introduction

Oral cancer (including cancers of the mouth, lip and tongue) is a major public health problem in certain regions of Europe, Latin America, Melanesia and Asia, including India [1], [2] where it ranks as one of the leading cancer sites among men and women in many regions [2]. Major risk factors for oral cancer (approximately half of which are located in the mouth [2]), are the use of tobacco, betel quid and alcohol [3], [4]. Intake of fruits and vegetables is thought to be protective [5].

Despite existing tobacco and alcohol control policies [6], [7], mouth cancer incidence in men has been increasing in most population-based cancer registries (PBCRs) in India (1.4 to 7.0%) [2]: five urban PBCRs reported a near doubling of mouth cancer cases between 1988–90 and 2003–05 among men with a relative change of 95.3% over these periods [8]. Although some PBCRs have shown decreasing rates of mouth cancer among women (−0.6 to −2.6%) [2], rates among women have remained largely unchanged for most Indian PBCR’s over a 30-year period (1980–2010) [2].

In a country such as India, where access to healthcare services and cancer-related awareness is highly variable, changes in incidence rates should be interpreted carefully. A more in-depth analysis of important underlying factors related to age, time period and birth cohort for these trends can yield information for planning rationale cancer control programmes. We conducted an age-period-cohort analysis (APC) of mouth cancer incidence trends using the PBCR data in Mumbai over a 15-year time period (1995–2009) to address the trends of one of the leading cancer sites in Mumbai, and to better understand the differences by gender. We focused on more recent time trends with a view to understanding whether the trend is allied to specific period and cohort effects that may reflect underlying lifestyle risk factors, and therefore improve and/or better target populations in existing cancer control programmes.

## 2 Methods

### 2.1 Incidence

The Mumbai Cancer Registry routinely collects data on all cancer cases residing in Mumbai for a duration of one year or more from 140 collaborating hospitals [9], [10]. Medical charts and pathology reports were reviewed to abstract clinical information including date and method of diagnosis; histological grade, subtype, and stage of disease; and demographic information such as age, gender, religion and marital status. Details of case registration are published in the Mumbai Cancer Registry Annual Report [11]. We extracted information on all incident cases of cancer of the mouth [ICD-O-3; gingiva (C03), floor of the mouth (C04), palate (C05), cheek mucosa, vestibule, retromolar area and other unspecified parts of the mouth (C06)] diagnosed between 1995 and 2009 from the Mumbai Cancer Registry.

### 2.2 Study population

The study population included male and female residents of Mumbai aged 25–74 years of age and diagnosed between 1995 and 2009. We excluded incident cases below 25 years and above 74 years of age due to paucity of incidence as well as less complete and accurate diagnostic data in older persons (75+ years). Subjects were categorised into ten 5-year age groups (25–29, 30–34, 35–39…70–74 years) and three 5-year calendar periods (1995–1999, 2000–2004, and 2005–2009) based on their respective age and year of diagnosis. Twelve 5-year birth cohorts (1925–1929, 1930–1934….1979–1984) were constructed by subtracting the 5-year age band from the corresponding 5-year period of diagnosis.

### 2.3 Population data

Annual population estimates were interpolated from decennial Census of India data (Census of India: Census population tables. Maharashtra part IIA 1991; Census of India: Series 28, Maharashtra Provisional population 2001; Census of India: Greater Mumbai 2011) [12]. Applying an average relative change in population per year and assuming a linear increase in population, the annual population for Mumbai, Maharashtra was estimated for each year from 1995 to 2009.

### 2.4 Statistical data

All analyses were carried out in men and women separately. Age-specific and age-standardized incidence rates (using the world standard-Segi 1960 [13] truncated for the age group 25–74) were calculated per 100,000 for each gender and by three 5-year calendar periods. A more formal assessment of the contribution of age, period and cohort effects involved the fitting of APC models to the trends [14], [15]. Overall goodness-of-fit tests as well as statistical tests for the contribution of the overall slope (net drift) and the effects of period and cohort curvature were obtained using the analysis of deviance of nested models, as suggested by Clayton and Schifflers [14], [15]. In this framework, the importance of non-linear period and cohort effects is statistically tested using the log-likelihood ratio test comparing the simple trend model (age-drift model) with the two-factor (age–period and age–cohort) models to evaluate nested models on the basis of the addition of cohort and period term. The final model was selected on the grounds of parsimony. The age-drift model was used to summarise the magnitude and direction of temporal trends over the period 1995–2009. The net drift parameter is a one-degree-of-freedom linear term for time that represents the estimated annual percent change (EAPC) in the rates over the passage of time that is common to calendar period and birth cohort [14], [15]. The EAPC is the average change in the trend over the designated time of study and is linear on a log scale and thus comparable, irrespective of the magnitude of the rates at baseline. Stata: 10 (StataCorp. 2010) was used for data management and analysis.

## 3 Results

The age-standardized incidence rates of mouth cancer in men aged 25–74 years in Mumbai, India increased linearly over the 15-year study period, with a mean estimated increase of 2.7% (95% CI: 1.9 to 3.4, p < 0.0001) per year (Table 1). In contrast, rates among women in the same age range decreased by 0.01% (95% CI: 0.02 to 0.002, p = 0.03) on average per year (Table 1).

**Fig. 1:**
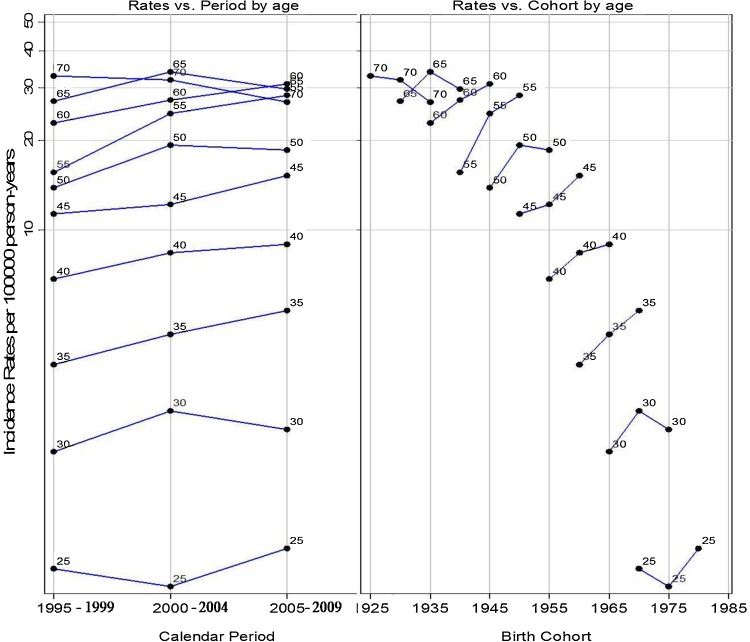
Observed rates of mouth cancer in men aged 25–74 and diagnosed between 1995 and 2009. Rates are plotted vs. calendar period and birth cohort for each age at diagnosis group.

The APC model revealed significant positive non-linear period and cohort effects in men, yielding the full APC model as the best fitting for mouth cancer trends for these data. The respective graphs of rates by time period and birth cohort indicated higher age-specific rates for younger men (aged 25–49 years) diagnosed in later time periods (2000–2009) and for those born in later cohorts across all age groups. The downward concavity in the cohort effects in ages between 30–34 years and 70–74 years (Fig. 1) suggests a slight deceleration in the rate of increase (Table 2). The slopes for both time period and birth cohort indicated that the increasing incidence trend was higher in younger (aged 25–49 years) than in older men (aged 50–74 years).

**Fig. 2:**
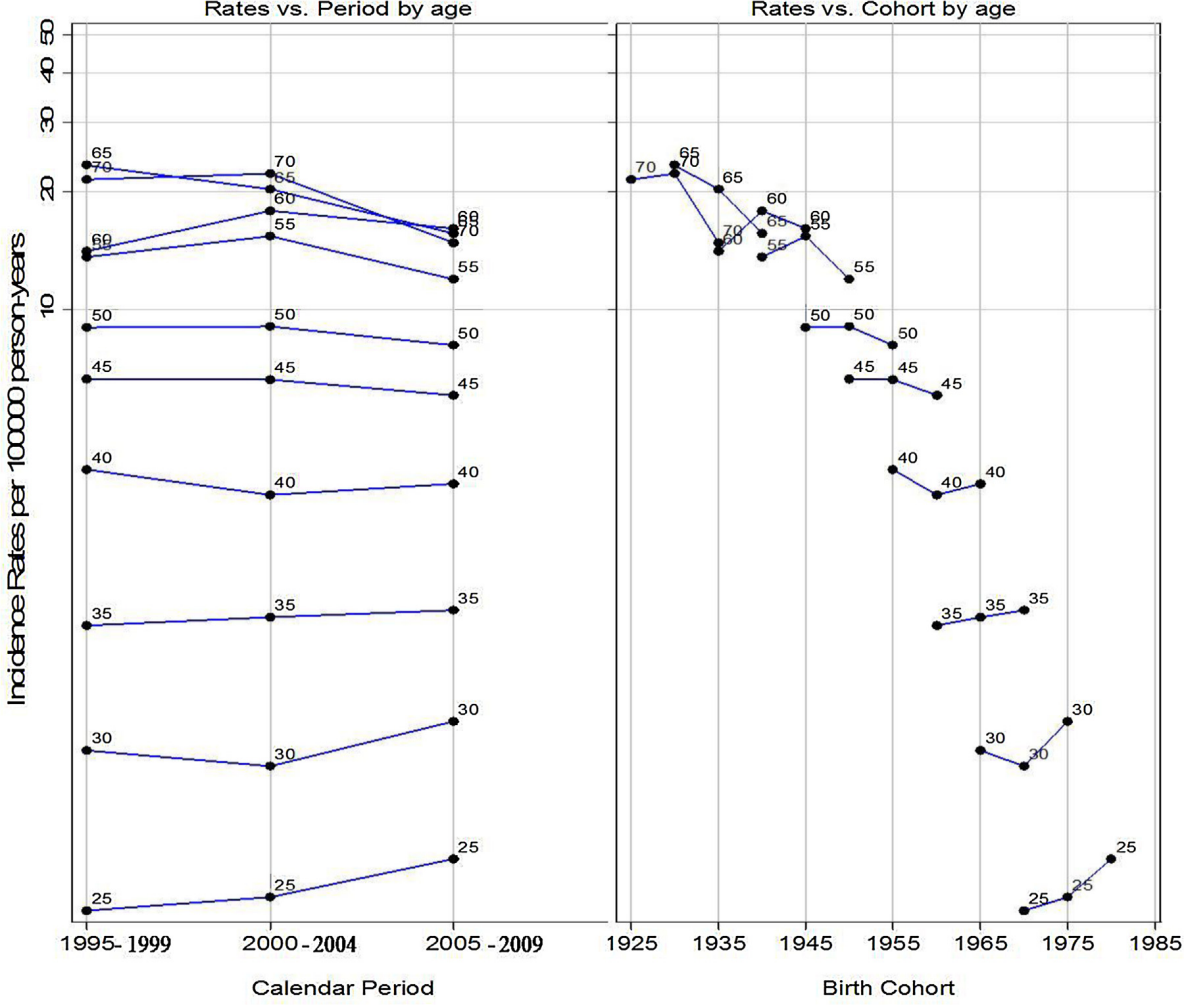
Observed rates of mouth cancer in women aged 25–74 and diagnosed between 1995 and 2009. Rates are plotted vs. calendar period and birth cohort for each age at diagnosis group.

The APC model analyses among women aged 25–74 in Mumbai (Table 3) indicated borderline significant (p = 0.06) negative period effects but no cohort effects. For women, the near-parallel lines exhibited between successive periods of diagnosis convey little with regards to the relative importance of period curvature. However, both period and birth cohort graphs for women indicated an increasing trend in younger women (aged 25–49) (Fig. 2) with period effects stronger than the cohort effects (Table 3). The overall borderline significant negative period effects in women further indicated that the decreasing rates in older groups (>49 years) counteracted the rising rates in younger women (aged 25–49 years) in the overall rates.

## 4 Discussion

An APC analysis of 15-year data for incidence of mouth cancer among men and women living in Mumbai indicated a significant annual 2.7% increase in men between 1995 and 2009, and a 0.01% annual decrease in women over the same time period. In men aged 25–74 years, we observed significant positive period and cohort effects through APC models, with the observed increase greater in younger men (25–49 years) and those who were born during later time periods (1975–1984). In women, a borderline significant overall negative period effect was observed, but increasing rates were observed in younger women (aged 25–49 years) across all periods.

Studies from PBCRs in India have largely reported similar trends among men and women [2], [16], [17], [18], [19] (Appendix A Table A1). NCRP data from Mumbai [2] show a steep increase of mouth cancer incidence in men from 1999 to 2009 (3.3% each year) and a slight increase among women from 2002 to 2009 [2]. The disability adjusted life years (DALYs) of mouth cancer in India is high (77 vs.46.9/100,000 globally), making it a key public health issue [20].

Although we cannot compare incidence trends for mouth cancer per se across different high incidence countries, since oral cancers (cancers of mouth, lip and tongue) are generally studied together [1], [2], overall oral cancer trends are likely to be similar to trends for mouth cancer. South Asian countries such as India, Sri Lanka, Pakistan, Bangladesh and Taiwan report some of the highest incidence rates (ASR) of oral cancer in the World [1] due to betel quid and tobacco chewing habits, coupled with low awareness and health care access, and poor referrals of diagnosis and care. While Sri Lanka in recent years has shown a decreasing trend of oral cancers of about 1.9% per year (p < 0.05) in both men and women [21], Taiwan [22] and Pakistan [19] have consistently showed increasing trends in both men and women. Brazil showed similar trends in men and women as in our study [23]. Among European countries with high incidence of oral cancer, rates in France and Slovakia have been decreasing among men and increasing among women [23], [24]. Oral cancer trends have been decreasing in both men and women in all other developed countries except United Kingdom, Denmark and Netherlands, which show increases in recent years [23], [25], [26] (Appendix A, Table A.2). The decline in oral cancer incidence trends in most parts of the world, especially high-income regions, is consistent with increased awareness and decline in tobacco use [23].

Increasing trends of oral cancer in certain high incidence regions including India can likely be attributed to increased risk exposures such as tobacco and betel quid chewing with synergistic alcohol effects which are unique to India and other high incidence countries [4], [23], [27]. This is further supported by reports that increases in oral cancer incidence in developed countries such as United Kingdom may partly be due to migrated South Asian populations [23].

Published results of house to house surveys in men and women aged ≥35 years (n = 99,598) in Mumbai city in 1992–94 revealed that about 69.3% of men were tobacco users out of which 45.7% were tobacco chewers [28]. According to National Family Health Surveys (NFHS-2 and 3), 60% of men in Maharashtra were daily users of tobacco/pan-masala and/or alcohol in 1998–99, 88.2% of men in Mumbai city were tobacco/pan-masala and/or alcohol users in 2005–6 [29]. The latest Global Adult Tobacco Survey (GATS 2009–10), among men aged 15 years and above, in the state of Maharashtra revealed that a majority of current tobacco users were daily tobacco chewers [30]. In the same period (1992–94), tobacco use in women was slightly less in Mumbai city (57.5% vs. 69.3% in men), but again tobacco chewing was the most prevalent form [28]. Subsequent years have seen a decrease in the prevalence of tobacco use among women, with only 19% of women in Maharashtra reporting tobacco/pan-masala use [29]. The latest GAT Survey (GATS 2009–10), however showed a rising prevalence of tobacco chewing among women aged ≥15years, with a large proportion reporting daily use (17.5%) [30].

We observed increasing trend of mouth cancer in younger cohorts of men and women. In our study population 40.1% (n = 1900) of total cases (n = 4735) between ages 25 and 74 in men and 31.3% (n = 658) of total cases (n = 2104) in women between ages 25 and 74 occurred between 25 and 49 years. Trend analysis over a long period of time (say here, 1995–2009; over a period of 15 years) compensates for the random variation which could occur when comparing rates annually/bi-annually, for a smaller sub-set of cases or for a shorter period. Additionally, age-period-cohort analysis is a sophisticated method which teases out the influence of ‘age’ on the trends over a long period of time. Thus, it is probable that what we observed in Mumbai population is the actual trend and unlikely to be due to random variation. This could further be explained by the high prevalence of tobacco use among youth and an early age of initiation. The Global Youth Tobacco Survey (GYTS-1999 & 2001) conducted among school children aged 13–15 years indicated that 9.6% were current users of tobacco in any form, out of which about 52.5% initiated tobacco use before 10 years of age [31]. The mean age at initiation of tobacco use in Maharashtra was 18.5 years and 12% of men and women were below fifteen. In India more women tend to start tobacco use before the age of 15 than men (25.8% vs. 13.2% in men) [30]. The fact that tobacco and betel quid use is prevalent among vulnerable populations (i.e. illiterate and lower SES) coupled with lower consumption of fruits and vegetables in men and women in Maharashtra could also have partially contributed to the increasing trend of mouth cancers in Mumbai [29].

While significant period effects can be caused by variations specific to a time period irrespective of age, such as the introduction of new diagnostics, screening programmes and greater awareness or availability of health services, these factors are not expected to play a major role in our observed trends given that we do not observe a systematic influence of these factors among both men and women. Sudden changes in the coding systems and completeness of registration can also create period effects, but it is unlikely that the effects we observed are due to changes in the registry system, since opposite directions of trend were observed in men and women, and also because the Mumbai cancer registry, the first PBCR established in India in 1963, has maintained reliable data on cancer [16], [17], [32] (90% of mouth cancer cases registered through microscopic confirmation [11]). It has consistently met IARC’s quality standards with respect to inclusion in consecutive volumes of their Cancer Incidence of Five Continents series (volumes II to IX) over this time [33], [34]. There has been no change in the area of the region covered by the cancer registry or the coding system during our entire study period.

Stringent tobacco control policies and programmes have been in place in India since May 2004 [6], including advertisements to be restricted only to point- of- sale, prohibition of sale of tobacco products to children less than 18 years of age and near educational institutions, health warnings, and declaration of product contents on packs [6]. With these recent developments in tobacco control, the incidence of mouth cancers was expected to decrease. However, to date the enforcement of these policies has been weak to moderate [7] which is reflected in the increasing trend observed in our study especially among young adults. Increased awareness regarding tobacco use and oral cancer risk has been recently reported among urban slum women of Mumbai [35], which could have an effect in future trends.

In spite of some limitations such as the inability to infer causal association [9], the APC model can be used to understand complex patterns in cancer trends when high-quality registry data are available on age, sex, date of diagnosis, tumour site and histology [9]. The rationale behind this modelling assumes that the increasing trends in mouth cancer are allied to generational influences which could be due to period changes or birth cohort per se or a changing prevalence and distribution of known lifestyle and environmental factors in men and women of Mumbai, and that these influences should, with a time lag, result in changing cancer rates observed in successive birth cohorts. The increasing trends of mouth cancer that we observed among men in Mumbai, and in both men and women aged 25–49 years underscore the public health importance of targeted programmes to decrease the prevalence of risk factors in young men and women, as India continues to observe increases in rates of mouth cancer.

## Conflict of interest

None declared.

## Ethical approval

Not required.

## Role of funding source

Funders had no role in the study design, data collection, analysis or publication of the manuscript.

## Figures and Tables

**Table 1:** Mouth cancer in Mumbai 1995–2009: person-years at risk, incidence cases, age-standardized rates, overall linear trends for men and women (aged 25–74).

Calendar period	MEN (aged 25–74 yrs)			WOMEN (aged 25–74)		
	Number of cases^a^	Person-years^a^ (millions)	ASR^b^	Number of cases^a^	Person-years^a^ (millions)	ASR^b^
1995–99	229.6	3.1	10.3	120.2	2.5	6.9
2000–04	322	3.4	12.8	144.2	2.7	7.2
2005–09	395.4	3.9	13.4	156.4	3.1	6.1
Estimated APC^c^ (95%CI) ​1995-2009	2.7 (1.9 to 3.4); p < 0.0001			−0.01 (−0.02 to −0.002); p = 0.03		

a Mean annual numbers.

b Age-standardized rate per 100,000 (world standard, Segi 1960).

c Estimated annual percentage change obtained from log-linear model, with 95% confidence interval (net drift).

**Table 2:** Mouth cancer in Mumbai 1995–2009, men aged 25–74. Analysis of deviance for nested APC models.

Model No.	Model description	Goodness-of-fit			Model Comparison	Effect Tested	Difference between models		
		df^a^	Residual Deviance	p			df^a^	Deviance	p
0	Age	9	149.8	<0.0001					
1	Age + drift	10	122.3	<0.0001	1 vs. 0	Drift	1	27.5	<0.0001
2	Age + period	11	118.8	<0.0001	2 vs.1	Non-linear period	1	3.5	0.0080
3	Age + cohort	20	107.7	<0.0001	3 vs.1	Non-linear cohort	10	14.6	0.0012
4	Age+ period + cohort^b^	21	103.2	<0.0001	4 vs. 3	Non-linear period^c^	1	4.5	0.002
					4 vs.2	Non-linear cohort^c^	10	15.6	0.0005

a Degrees of freedom.

b Best-fitting APC model on the grounds of significant non-linear period and cohort effects.

c Period (cohort) adjusted for non-linear cohort (period).

**Table 3:** Mouth cancer in Mumbai 1995–2009, women aged 25–74. Analysis of deviance for nested APC models.

Model No.	Model description	Goodness-of-fit			Model Comparison	Effect Tested	Difference between models		
		df^a^	Residual Deviance	p(>∣chi∣)			df^a^	Deviance	p(>∣chi∣)
0	Age	9	100.3	<0.0001					
1	Age + drift	10	97.9	<0.0001	0 vs. 1	Drift	1	2.4	0.03
2	Age + period	11	96.1	<0.0001	2 vs.1	Non-linear period	1	1.8	0.06
3	Age + cohort	20	92.2	<0.0001	3 vs.1	Non-linear cohort	10	5.7	0.32
4	Age+ period + cohort^b^	21	90.1	<0.0001	4 vs. 3	Non-linear period^c^	1	2.1	0.94
					4 vs.2	Non-linear cohort^c^	10	6.0	0.29

a Degrees of freedom.

b Best-fitting APC model on the grounds of parsimony.

c Period (cohort) adjusted for non-linear cohort (period).
