# Supplementary material for: Trends in mouth cancer incidence in Mumbai, India (1995–2009): An age-period-cohort analysis
Source: Cancer Epidemiol. 2016 Jun;42:66–71. doi: 10.1016/j.canep.2016.03.007 (PMC4911594; doi:10.1016/j.canep.2016.03.007)
Supplement: Supplementary file 1 [file mmc1.docx]

Join-point analyses for trends in mouth cancer incidence in Mumbai, India (1995-2009)

We conducted join-point regression analysis for the data. The figures for join-point analyses are presented below:
  MEN (for all ages)


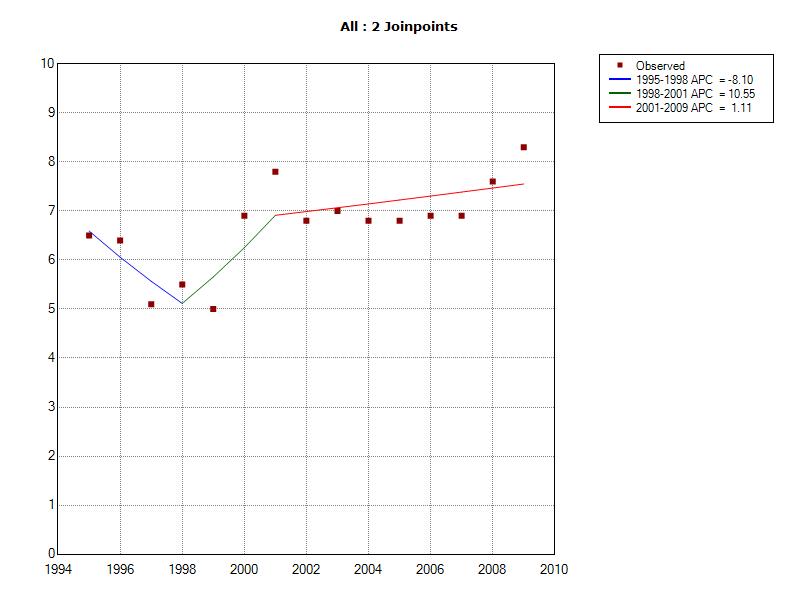


Age Standardized Incidence Rates

Period of Diagnosis

In men, there was a dip in the incidence rates between 1995 and 1998 but a steep rise between 1998 and 2001 followed by gradual increase in the incidence rates from 2001 to 2009.


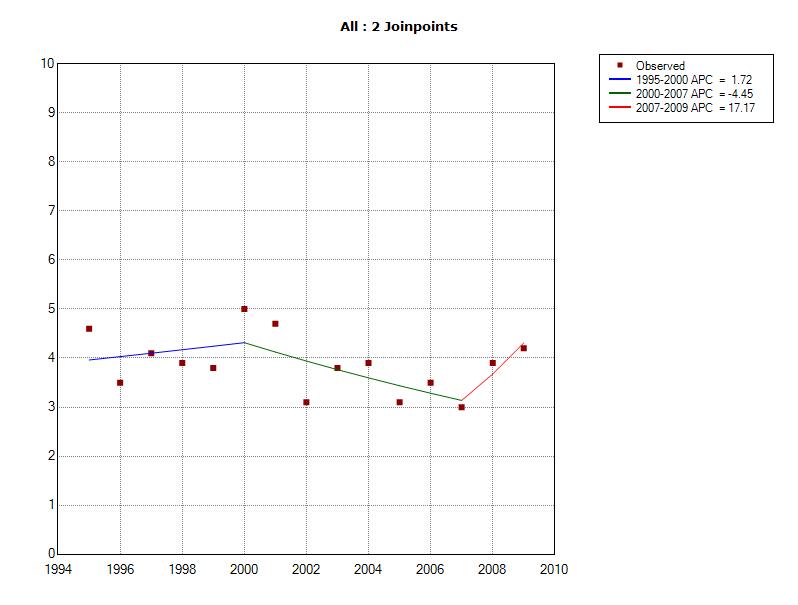
 WOMEN (for all ages)

Period of Diagnosis

Age Standardized Incidence Rates

Among women we observed a nearly stable incidence rates between 1995 and 2000 followed by gradual decrease (2000-2007) and an increase in the incidence rates from 2007 to 2009.
