# Supplementary material for: Trends in mouth cancer incidence in Mumbai, India (1995–2009): An age-period-cohort analysis
Source: Cancer Epidemiol. 2016 Jun;42:66–71. doi: 10.1016/j.canep.2016.03.007 (PMC4911594; doi:10.1016/j.canep.2016.03.007)
Supplement: Supplementary file 2 [file mmc2.doc]

**APPENDIX TABLE A.1:** Mouth Cancer Trends in Population Based Cancer Registries of India

| **N** | **Author/Year** | **Expected Annual Percent Change (%)** | | **Period and Cohort effects** | **Method** | **Data Source/ Year** |
| --- | --- | --- | --- | --- | --- | --- |
|  |  | **MEN** | **WOMEN** |  |  |  |
| 1 | Shridhar et al, 2014  (current study) | 2.7  (1.9 to 3.4) | -0.01  (-0.02 to -0.002) | Significant in men  Effects greater in younger men and women (25-49 yrs) | APC | Mumbai  1995 to 2009 |
| 2 | Chaturvedi et al,  2013* [1] | 1.0  (0.4 to 1.7) | -2.1  (-1.4 to -2.8) | NR | APC | Country specific (CI5)  1983 to 2002 |
| 3 | NCRP, 2013 | 1.4 (Mumbai) to 7.0 (Dibrugarh) | -0.6 (Mumbai) to  -2.6 (Chennai) | NA | Join-point regression | Individual PBCRs  1980s to 2009^ρ^ |
| 4 | Yeole et al, 2007 [3] | -1.8 (Bangalore)  0.75 (Mumbai) | 3.4 (Delhi)  2.8 (Chennai)  -3.3 (Bangalore) | NA | Linear regression | Mumbai, Bangalore Chennai, Delhi, Bhopal, Barshi (1980s to 2003) ^ρ^ |
| 5 | Elango et al, 2006 [4] | NR | NR | NA | Linear regression | Chennai , Barshi  1986 to 1988 |
| 6 | Sunny et al,  2004** [5] | -1.7 | NS | NA | Linear regression | Mumbai 1986-2000 |

NR-Not Reported; NA-Not Applicable; CI5-Cancer Incidence of 5 Continents; ρ- time period varies according to the available data; APC- age-period-cohort *mouth and oral tongue; **lip, tongue, mouth; NS-Not Significant

**APPENDIX TABLE A.**2: COMPARISON OF MUMBAI INCIDENCE TRENDS WITH OTHER HIGH INCIDENCE COUNTRIES

|  | | ASR (2012)* | TREND | | COMMENTS |
| --- | --- | --- | --- | --- | --- |
| Mumbai**  (current study) | Men | 8.2 |  | | Period and cohort effects significant in men. Effects greater in younger men and women (1995-2009) |
|  | Women | 4.2 |  | |  |
| Papua New Guinea | Men | 30.3 |  | | No data for New Guinea but Pacific islands in general and Melanesia in particular( 1997 and 2007 %relative change) [1] |
|  | Women | 21.1 |  | |  |
| Sri Lanka | Men | 15.5 |  | | Based on log-linear regression of hospital-based cancer registries 1985-2005 [6] |
|  | Women | 5.7 |  | |  |
| Taiwan | Men | 16.1† |  | | Based on log-linear regression of National cancer registry data 1979-2007 [7] |
|  | Women |  |  | |  |
| Pakistan | Men | 10.5  9.1 |  | | Based on log-linear regression of Karachi Cancer Registry 1995-2004 [8] |
|  | Women |  |  | |  |
| Bangladesh | Men | 13.0 |  | | No data |
|  | Women | 5.9 |  | |  |
| France | Men | 13.7 |  | | Based on APC of data from CI5 1983-2002 [1] |
|  | Women | 2.7 |  | |  |
| Slovakia | Men | 11.4 |  | | Based on APC of data from CI5 1983-2002 [1] |
|  | Women | 2.4 |  | |  |
| Hungary | Men | 15.5 |  | | Based on join-point regression of WHO data 1975-2004 [9] |
|  | Women | 4.6 |  | |  |
| **India** | Men | 10.1 |  | | Based on join-point regression model of NCRP 2013 1980s-2009 . Significant decreasing trend reported only from Bangalore and Chennai PBCRs among women |
|  | Women | 4.3 | Unchanged/ | |  |
| Slovenia | Men | 7.6 |  | | Based on APC of data from Slovenia cancer registry predicted upto 2009 [10] |
|  | Women | 2.7 |  | |  |
| Brazil | Men | 7.2 |  | | Based on APC of data from CI5 1983-2002 [1] |
|  | Women | 2.9 |  | |  |
| **Other developed countries** | | | | | |
| UK | Men | 6.2 | |  | Based on APC of data from CI5 1983-2002 [1] |
|  | Women | 3.2 | |  |  |
| Denmark | Men | 6.9 | |  | Based on APC of data from CI5 1983-2002 [1] |
|  | Women | 3.9 | |  |  |
| Netherlands | Men | 5.6 | |  | Based on APC of data from CI5 1983-2002 [1] |
|  | Women | 3.3 | |  |  |
| Spain | Men | 7.4 | |  | Based on APC of data from CI5 1983-2002 [1] |
|  | Women | 3.3 | |  |  |
| Italy | Men | 4.1 | |  | Based on APC of data from CI5 1983-2002 [1] |
|  | Women | 2.1 | |  |  |

*Globocan 2012 for lip and oral cavity except for Mumbai; ** ASR 2009 for mouth;

† Globocan 2008; APC- age-period-cohort analysis; CI5- Cancer Incidence of 5 continents

**References**

1. Chaturvedi AK, Anderson WF, Lortet-Tieulent J, Curado MP, Ferlay J, Franceschi S, Rosenberg PS, Bray F, and Gillison ML, Worldwide trends in incidence rates for oral cavity and oropharyngeal cancers. J Clin Oncol, 2013. **31**(36): 4550-9.

2. NCRP, Three year report of Population Based Cancer Registries, 2009-2011,Indian Council of Medical Research, National Cancer Registry Program: Bangalore. 2013

3. Yeole BB, Trends in incidence of head and neck cancers in India. Asian Pac J Cancer Prev, 2007. **8**(4): 607-12.

4. Elango JK, Gangadharan P, Sumithra S, and Kuriakose MA, Trends of head and neck cancers in urban and rural India. Asian Pac J Cancer Prev, 2006. **7**(1): 108-12.

5. Sunny L, Yeole BB, Hakama M, Shiri R, Sastry PS, Mathews S, and Advani SH, Oral cancers in Mumbai, India: a fifteen years perspective with respect to incidence trend and cumulative risk. Asian Pac J Cancer Prev, 2004. **5**(3): 294-300.

6. Ariyawardana A and Warnakulasuriya S, Declining oral cancer rates in Sri Lanka: are we winning the war after being at the top of the cancer league table? Oral Dis, 2011. **17**(7): 636-41.

7. Tseng CH, Oral cancer in Taiwan: is diabetes a risk factor? Clin Oral Investig, 2013. **17**(5): 1357-64.

8. Bhurgri Y, Cancer of the oral cavity - trends in Karachi South (1995-2002). Asian Pac J Cancer Prev, 2005. **6**(1): 22-6.

9. Garavello W, Bertuccio P, Levi F, Lucchini F, Bosetti C, Malvezzi M, Negri E, and La Vecchia C, The oral cancer epidemic in central and eastern Europe. Int J Cancer. **127**(1): 160-71.

10. Pompe-Kirn V, Japelj B, and Primic-Zakelj M, Future trends in breast, cervical, lung, mouth and pharyngeal cancer incidence in Slovenia. Cancer Causes Control, 2000. **11**(4): 309-18.
